# Supplementary material for: Polyketide synthase gene clusters in the ericoid mycorrhizal fungus Oidiodendron maius: genome-wide prediction, expression profiling, and functional characterization of knockout mutants
Source: Front Plant Sci. 2026 Sep 16;17:1924430. doi: 10.3389/fpls.2026.1924430 (PMC13623903; doi:10.3389/fpls.2026.1924430)
Supplement: Supplementary file 1 [file SupplementaryFile1.zip › Supplementary Materials/Supplementary Folder 1.zip!/Oidma1_AssemblyScaffolds_Repeatmasked/knownclusterblast/cluster45/jgi.p_Oidma1_135710_mibig_hits.html]

| MIBiG Protein | Description | MIBiG Cluster | MiBiG Product | % ID | % Coverage | BLAST Score | E-value |
| --- | --- | --- | --- | --- | --- | --- | --- |
| EAU38788.1 | conserved\_hypothetical\_protein | BGC0000161 | Terrein\_biosynthetic\_gene\_cluster | 62 | 99.3333333333 | 788 | 5e-228 |
| CBW45655.1 | putative\_oxygenase | BGC0000952 | Pristinamycin\_biosynthetic\_gene\_cluster | 32 | 59.8333333333 | 176 | 6.8e-44 |
| CAE17550.1 | oxygenase | BGC0000210 | Chromomycin\_A3\_biosynthetic\_gene\_cluster | 31 | 62.6666666667 | 169 | 1.4e-41 |
| ADE34483.1 | SsfO2 | BGC0000269 | SF2575\_biosynthetic\_gene\_cluster | 28 | 80.6666666667 | 165 | 2.7e-40 |
| AEI98648.1 | CtcE | BGC0000209 | Chlortetracycline\_biosynthetic\_gene\_cluster | 29 | 77.8333333333 | 161 | 3e-39 |
| AFU65903.1 | DacO2 | BGC0000216 | Dactylocycline\_biosynthetic\_gene\_cluster | 30 | 59.8333333333 | 160 | 8.6e-39 |
| AAZ78335.1 | OxyL | BGC0000254 | Oxytetracycline\_biosynthetic\_gene\_cluster | 28 | 80.8333333333 | 157 | 5.6e-38 |
| CAK50777.1 | oxygenase | BGC0000247 | Mithramycin\_biosynthetic\_gene\_cluster | 28 | 60.5 | 154 | 3.6e-37 |
| CAA07757.1 | oxygenase | BGC0000246 | Mithramycin\_biosynthetic\_gene\_cluster | 28 | 60.5 | 154 | 3.6e-37 |
| AKD43496.1 | Monooxygenase | BGC0001409 | Dutomycin\_biosynthetic\_gene\_cluster | 28 | 59.5 | 153 | 8.1e-37 |
| AEF32090.1 | monooxygenase | BGC0001333 | BE-54017\_biosynthetic\_gene\_cluster | 29 | 60.8333333333 | 145 | 2.2e-34 |
| AEO12714.1 | flavin\_monooxygenase | BGC0001334 | Cladoniamide\_biosynthetic\_gene\_cluster | 29 | 60.6666666667 | 141 | 3.2e-33 |
| BAQ25478.1 | monooxygenase | BGC0001288 | Maklamicin\_biosynthetic\_gene\_cluster | 30 | 61.5 | 140 | 5.4e-33 |
| ABX71099.1 | Lct16 | BGC0000238 | Lactonamycin\_biosynthetic\_gene\_cluster | 29 | 59.1666666667 | 139 | 9.3e-33 |
| AKQ20687.1 | monooxygenase | BGC0001337 | Lazarimide\_biosynthetic\_gene\_cluster | 29 | 60.3333333333 | 139 | 1.2e-32 |
| ACN64853.1 | PokO2 | BGC0001061 | Polyketomycin\_biosynthetic\_gene\_cluster | 28 | 60.3333333333 | 139 | 1.6e-32 |
| AAL33754.1 | putative\_monooxygenase/hydroxylase | BGC0000421 | Safracin\_biosynthetic\_gene\_cluster | 27 | 59.1666666667 | 128 | 3.6e-29 |
| CAO98853.1 | monooxygenase\_AufJ | BGC0000023 | Aurafuron\_biosynthetic\_gene\_cluster | 28 | 59.6666666667 | 128 | 3.6e-29 |
| ADI71441.1 | putative\_oxygenase | BGC0000203 | BE-7585A\_biosynthetic\_gene\_cluster | 27 | 67.3333333333 | 127 | 6.2e-29 |
| CAH10119.1 | putative\_oxygenase | BGC0000268 | Sch47554\_/\_Sch47555\_biosynthetic\_gene\_cluster | 26 | 72.3333333333 | 125 | 2.4e-28 |
| ADQ55479.1 | FAD-binding\_monooxygenase | BGC0000350 | ET-743\_biosynthetic\_gene\_cluster | 25 | 59.3333333333 | 125 | 2.4e-28 |
| AAZ78342.1 | OxyS | BGC0000254 | Oxytetracycline\_biosynthetic\_gene\_cluster | 28 | 63.5 | 124 | 5.3e-28 |
| CAM34363.1 | putative\_FAD-binding\_monooxygenase,\_PheA/TfdB\_family | BGC0000242 | Lysolipin\_biosynthetic\_gene\_cluster | 29 | 62.8333333333 | 124 | 5.3e-28 |
| AAM33676.1 | putative\_FAD-dependent\_oxidoreductase\_GrhO9 | BGC0000230 | Griseorhodin\_biosynthetic\_gene\_cluster | 28 | 61.5 | 123 | 6.9e-28 |
| EHM27503.1 | monooxygenase\_FAD-binding | BGC0000235 | Kiamycin\_biosynthetic\_gene\_cluster | 26 | 73.3333333333 | 122 | 2e-27 |
| WP\_030269258.1 | monooxygenase | BGC0001308 | Simocyclinone\_D9\_/\_simocyclinone\_D10\_/\_simocyclinone\_D11 | 26 | 72.3333333333 | 120 | 5.8e-27 |
| AEE65484.1 | monooxygenase | BGC0000223 | Fluostatin\_biosynthetic\_gene\_cluster | 28 | 60.1666666667 | 119 | 1.3e-26 |
| ABI22123.1 | monooxygenase | BGC0000422 | Saframycin\_A\_biosynthetic\_gene\_cluster | 34 | 38.6666666667 | 119 | 1.3e-26 |
| ACP19351.1 | SaqE | BGC0000267 | Saquayamycin\_Z\_/\_galtamycin\_B\_biosynthetic\_gene\_cluster | 27 | 59.0 | 119 | 1.7e-26 |
| ADE22302.1 | putative\_FAD-binding\_monooxygenase | BGC0000279 | Xantholipin\_biosynthetic\_gene\_cluster | 26 | 61.6666666667 | 119 | 1.7e-26 |
| ctg1\_orf39 |  | BGC0001013 | Meridamycin\_biosynthetic\_gene\_cluster | 29 | 60.5 | 117 | 4.9e-26 |
| AEI98657.1 | CtcN | BGC0000209 | Chlortetracycline\_biosynthetic\_gene\_cluster | 28 | 63.6666666667 | 116 | 8.4e-26 |
| ADB02837.1 | AzicO3 | BGC0000202 | Azicemicin\_biosynthetic\_gene\_cluster | 26 | 68.1666666667 | 116 | 8.4e-26 |
| BAV16997.1 | putative\_oxygenase | BGC0001384 | Saprolmycin\_biosynthetic\_gene\_cluster | 24 | 72.5 | 116 | 8.4e-26 |
| AAM97364.1 | RubN | BGC0000266 | Rubromycin\_biosynthetic\_gene\_cluster | 27 | 59.3333333333 | 115 | 1.9e-25 |
| AKD43495.1 | Monooxygenase | BGC0001409 | Dutomycin\_biosynthetic\_gene\_cluster | 28 | 60.6666666667 | 115 | 1.9e-25 |
| ACN64854.1 | PokO4 | BGC0001061 | Polyketomycin\_biosynthetic\_gene\_cluster | 28 | 60.6666666667 | 115 | 2.4e-25 |
| AAL15585.1 | Sim7 | BGC0000270 | Simocyclinone\_biosynthetic\_gene\_cluster | 29 | 64.0 | 114 | 3.2e-25 |
| BAJ07857.1 | putative\_oxygenase | BGC0000232 | Hatomarubigin\_biosynthetic\_gene\_cluster | 31 | 43.5 | 114 | 4.2e-25 |
| AAD13534.1 | oxygenase\_homolog | BGC0000239 | Landomycin\_biosynthetic\_gene\_cluster | 26 | 67.3333333333 | 114 | 4.2e-25 |
| CAA60567.1 | putative\_oxygenase | BGC0000276\_c4 | Urdamycin\_biosynthetic\_gene\_cluster | 24 | 67.5 | 114 | 4.2e-25 |
| ABC02791.1 | putative\_FAD-monooxygenase | BGC0000809 | AT2433\_biosynthetic\_gene\_cluster | 26 | 61.1666666667 | 113 | 1.2e-24 |
| AGZ15446.1 | putative\_polyketide\_oxygenase/hydroxylase | BGC0001036 | Polyoxypeptin\_biosynthetic\_gene\_cluster | 27 | 61.5 | 111 | 2.7e-24 |
| AAG30198.1 | oxygenase | BGC0000261 | R1128\_biosynthetic\_gene\_cluster | 25 | 68.3333333333 | 110 | 6e-24 |
| WP\_030269274.1 | oxidoreductase | BGC0001308 | Simocyclinone\_D9\_/\_simocyclinone\_D10\_/\_simocyclinone\_D11 | 28 | 59.8333333333 | 110 | 7.9e-24 |
| ACP19358.1 | SaqM | BGC0000267 | Saquayamycin\_Z\_/\_galtamycin\_B\_biosynthetic\_gene\_cluster | 27 | 65.0 | 110 | 7.9e-24 |
| AAK57522.1 | PgaE | BGC0000262 | Rabelomycin\_biosynthetic\_gene\_cluster | 25 | 67.3333333333 | 108 | 2.3e-23 |
| CAD19095.1 | cytochrome\_P450\_dependent\_monooxygenase | BGC0000153 | Stigmatellin\_biosynthetic\_gene\_cluster | 26 | 57.8333333333 | 108 | 2.3e-23 |
| ADE22300.1 | putative\_FAD-binding\_monooxygenase | BGC0000279 | Xantholipin\_biosynthetic\_gene\_cluster | 25 | 62.5 | 108 | 3e-23 |
| ADB02842.1 | AzicO4 | BGC0000202 | Azicemicin\_biosynthetic\_gene\_cluster | 27 | 60.6666666667 | 107 | 5.1e-23 |
| CBH32087.1 | putative\_FAD-dependent\_monooxygenase | BGC0000211 | Chrysomycin\_biosynthetic\_gene\_cluster | 28 | 60.5 | 106 | 8.7e-23 |
| AAP69582.1 | putative\_FAD-dependent\_oxygenase | BGC0000226 | Gilvocarcin\_biosynthetic\_gene\_cluster | 27 | 60.8333333333 | 106 | 1.5e-22 |
| AAK06782.1 | putative\_oxygenase\_SimA7 | BGC0001072 | Simocyclinone\_biosynthetic\_gene\_cluster | 25 | 67.1666666667 | 106 | 1.5e-22 |
| ABP54640.1 | monooxygenase,\_FAD-binding | BGC0000241 | Lomaiviticin\_biosynthetic\_gene\_cluster | 25 | 59.5 | 104 | 3.3e-22 |
| AAM33673.1 | putative\_FAD-dependent\_monooxygenase\_GrhO6 | BGC0000230 | Griseorhodin\_biosynthetic\_gene\_cluster | 27 | 59.0 | 102 | 1.6e-21 |
| AHZ61860.1 | Lom26 | BGC0000240 | Lomaiviticin\_biosynthetic\_gene\_cluster | 27 | 61.8333333333 | 102 | 2.1e-21 |
| ABC87527.1 | FAD-dependent\_monooxygenase | BGC0001011 | Meridamycin\_biosynthetic\_gene\_cluster | 29 | 53.0 | 102 | 2.1e-21 |
| AAK06789.1 | putative\_oxygenase\_SimA8 | BGC0001072 | Simocyclinone\_biosynthetic\_gene\_cluster | 28 | 64.0 | 102 | 2.1e-21 |
| CBH32810.1 | putative\_oxygenase | BGC0000263 | Ravidomycin\_biosynthetic\_gene\_cluster | 27 | 60.0 | 101 | 2.8e-21 |
| ABP54639.1 | monooxygenase,\_FAD-binding | BGC0000241 | Lomaiviticin\_biosynthetic\_gene\_cluster | 25 | 68.3333333333 | 99 | 1.1e-20 |
| AHZ61851.1 | Lom17 | BGC0000240 | Lomaiviticin\_biosynthetic\_gene\_cluster | 26 | 59.1666666667 | 98 | 2.4e-20 |
| AEF32100.1 | RebC-like\_monooxygenase | BGC0001333 | BE-54017\_biosynthetic\_gene\_cluster | 24 | 78.0 | 97 | 5.3e-20 |
| AHZ61850.1 | Lom16 | BGC0000240 | Lomaiviticin\_biosynthetic\_gene\_cluster | 29 | 32.6666666667 | 96 | 1.2e-19 |
| AKQ20690.1 | RebC-like\_monooxygenase | BGC0001337 | Lazarimide\_biosynthetic\_gene\_cluster | 25 | 60.8333333333 | 94 | 5.8e-19 |
